# Phylogenetic characterization of Orthobunyaviruses isolated from Trinidad shows evidence of natural reassortment

**DOI:** 10.1007/s11262-023-01973-5

**Published:** 2023-02-10

**Authors:** Jerome E. Foster, Krisangel López, Gillian Eastwood, Hilda Guzman, Christine V. F. Carrington, Robert B. Tesh, Albert J. Auguste

**Affiliations:** 1 Department of Preclinical Sciences, Faculty of Medical Sciences, The University of the West Indies, St. Augustine, Republic of Trinidad and Tobago; 2 Department of Entomology, College of Agriculture and Life Sciences, Fralin Life Science Institute, Virginia Polytechnic Institute and State University, Blacksburg, VA 24061 USA; 3 Center for Emerging, Zoonotic, and Arthropod-Borne Pathogens, Virginia Polytechnic Institute and State University, Blacksburg, VA 24061 USA; 4 Global Change Center at Virginia Tech, Blacksburg, VA 24061 USA; 5 Department of Pathology, University of Texas Medical Branch, Galveston, TX 77555 USA

**Keywords:** *Orthobunyavirus*, Arbovirus ecology, Arbovirus evolution, Reassortment, Mosquito surveillance

## Abstract

The genus *Orthobunyavirus* is a diverse group of viruses in the family *Peribunyaviridae*, recently classified into 20 serogroups, and 103 virus species. Although most viruses within these serogroups are phylogenetically distinct, the absence of complete genome sequences has left several viruses incompletely characterized. Here we report the complete genome sequences for 11 orthobunyaviruses isolated from Trinidad, French Guiana, Guatemala, and Panama that were serologically classified into six serogroups and 10 species. Phylogenetic analyses of these 11 newly derived sequences indicate that viruses belonging to the Patois, Capim, Guama, and Group C serocomplexes all have a close genetic origin. We show that three of the 11 orthobunyaviruses characterized (belonging to the Group C and Bunyamwera serogroups) have evidence of histories of natural reassortment through the M genome segment. Our data also suggests that two distinct lineages of Group C viruses concurrently circulate in Trinidad and are transmitted by the same mosquito vectors. This study also highlights the importance of complementing serological identification with nucleotide sequencing when characterizing orthobunyaviruses.

**Supplementary Information:**

The online version contains supplementary material available at 10.1007/s11262-023-01973-5.

## Introduction

The family *Peribunyaviridae* comprises four genera including the *Orthobunyaviruses*, *Herbeviruses*, *Pacuviruses*, and *Shangaviruses*. Among these genera, the genus *Orthobunyavirus* possesses significant strain and genetic diversity, with approximately 88 recognized members [1], of which, at least 30 are known vertebrate pathogens [2]. Orthobunyaviruses are among the most widely distributed groups of arboviruses [3], and can be transmitted by a variety of vectors including mosquitoes, ticks, Culicoides midges, and phlebotomine sandflies [4].

Orthobunyaviruses have a tripartite negative (−) sense RNA genome consisting of L (large), M (medium), and S (small) RNA segments [5]. The L segment encodes the RNA-dependent RNA polymerase necessary for RNA replication. The M segment encodes the viral surface glycoproteins, Gn and Gc, and a nonstructural protein, NSm, that may play role in virus replication and pathogenesis [6, 7]. The S segment encodes a nucleocapsid protein (NP) and may include a small nonstructural protein, NSs, responsible for vertebrate immune evasion [8, 9]. Genome reassortment among and within *orthobunyavirus* species have been documented in the field and demonstrated experimentally [10–17].

Members of the genus are presently classified into ~ 18 serogroups [1], however, many closely related orthobunyaviruses within the same serogroup or species complex may be phylogenetically very distinct [14, 18]. These may present with small differences in antigenic properties, vectors, host range, pathogenicity, and uncertainty in their taxonomic relationships. Herein, we report the complete genome sequences and phylogenetic characteristics for 11 serologically identified orthobunyaviruses isolated from Trinidad, French Guiana, Guatemala, and Panama between 1954 and 2009.

## Materials and methods

Viruses were obtained from a two-year mosquito surveillance study conducted in Trinidad [19, 20], and historical isolates were obtained from the World Reference Center for Emerging Viruses and Arboviruses, at the University of Texas Medical Branch, Galveston, TX. All viruses included in this study were serologically identified by hemagglutination inhibition tests as previously described [19, 20]. Viruses were previously isolated using cytopathic effect (CPE) assays in Vero cells, propagated once more in Vero-76 cells, and concentrated by polyethylene glycol precipitation as previously described [21, 22] for RNA isolation. RNA was extracted using TRIzol LS (Invitrogen, Carlsbad, CA, USA) following the manufacturer’s protocol, and eluted in 30 µl of water and stored at – 80 ℃. Viral RNA was sequenced on an Illumina HiSeq 1000 (Illumina Inc. San Diego, CA, USA) using a paired-end 50 base sequencing by synthesis run. Viral contigs were assembled de novo using AbySS software [23], and confirmed using bowtie2 to align reads to the contigs [24]. Reads were visualized using the integrative genomics viewer [25].

Meta-data for the viruses sequenced in the study are presented in Table 1. Given the high level of sequence diversity observed among the orthobunyaviruses [26], analyses were performed on both the nucleotide and predicted amino acid sequences. All newly derived complete genome segment sequences were separately aligned using the ClustalW algorithm implemented in GENEIOUS v7.14 [27], and then manually aligned in the same software package with previously published sequences available from GenBank. Where possible, the majority of the known taxonomically named viruses within a species- or sero-complex was included in the phylogenetic analysis. The final data sets consisting of 82, 84, and 87 sequences for the S, M, and L segment open reading frames (ORFs), respectively, were down-sampled preferentially including named viruses that shared genetic relatedness to the viruses sequenced in this study. Alignments were trimmed to include only ORFs for analyses.

**Table 1:** *Orthobunyavirus* species sequenced in this study and associated meta-data

Virus name	Serocomplex	Isolate	Location (country)	Date (year of isolation)	Source	Accession number
Caraparu virus (CARV)	Group C	TRI712	Trinidad	2007	*Culex portesi*	L: OP931879 M: OP931890 S: OP931901
Caraparu-like virus (CARLV)^#^	Group C	TRVL34053	Trinidad	1960	Sentinel mice	L: OP931881 M: OP931892 S: OP931903
Caraparu-like virus (CARLV)^#^	Group C	TRI7121	Trinidad	2008	*Culex portesi*	L: OP931880 M: OP931891 S: OP931902
Guama virus (GMAV)	Guama	TRVL25714	Trinidad	1966	*Culex portesi*	L: OP931882 M: OP931893 S: OP931904
Inini virus (INIV)	Simbu	Cayan1093A	French Guiana	1973	Toucan sp.	L: OP931883 M: OP931894 S: OP931905
Melao virus (MELV)	California	TRVL9375	Trinidad	1955	*Aedes scapularis*	L: OP931884 M: OP931895 S: OP931906
Nepuyo virus (NEPV)	Group C	TRVL18462	Trinidad	1957	*Culex accelerans*	L: OP931885 M: OP931896 S: OP931907
Oriboca virus (ORIV)	Group C	TRI5972	Trinidad	2008	*Culex portesi*	L: OP931886 M: OP931897 S: OP931908
Ossa virus (OSSAV)	Group C	BT1820	Panama	1961	Human	L: OP931887 M: OP931898 S: OP931909
Shark River virus (SRV)	Patois	68U214	Guatemala	1968	*Melanochromis auratus*	L: OP931888 M: OP931899 S: OP931910
Wyeomyia virus (WYOV)	Bunyamwera	TRI5314	Trinidad	2009	*Trichoprosopon digitatum*	L: OP931889 M: OP931900 S: OP931911

^#^Unassigned species

Maximum likelihood (ML) phylogenetic trees were constructed for each amino acid dataset using Iq-Tree [28] under the best-fit nucleotide substitution model (i.e., for the L segment–LG + F + R5; for the M segment–LG + F + I + G4; for the S segment–LG + I + G4) selected by ModelFinder [29]. Ultrafast bootstrap (UFBoot [30]) with 1000 bootstrap samples were performed to assess the robustness of tree topologies. The consensus tree for each gene segment was then visualized, midpoint rooted, and annotated using Figtree (version 1.4.4). Nodes with UFBoot ≥ 95% were considered strongly supported. Sequence identities were also calculated using GENEIOUS v7.14 [27].

## Results and discussion

**Fig. 1:**
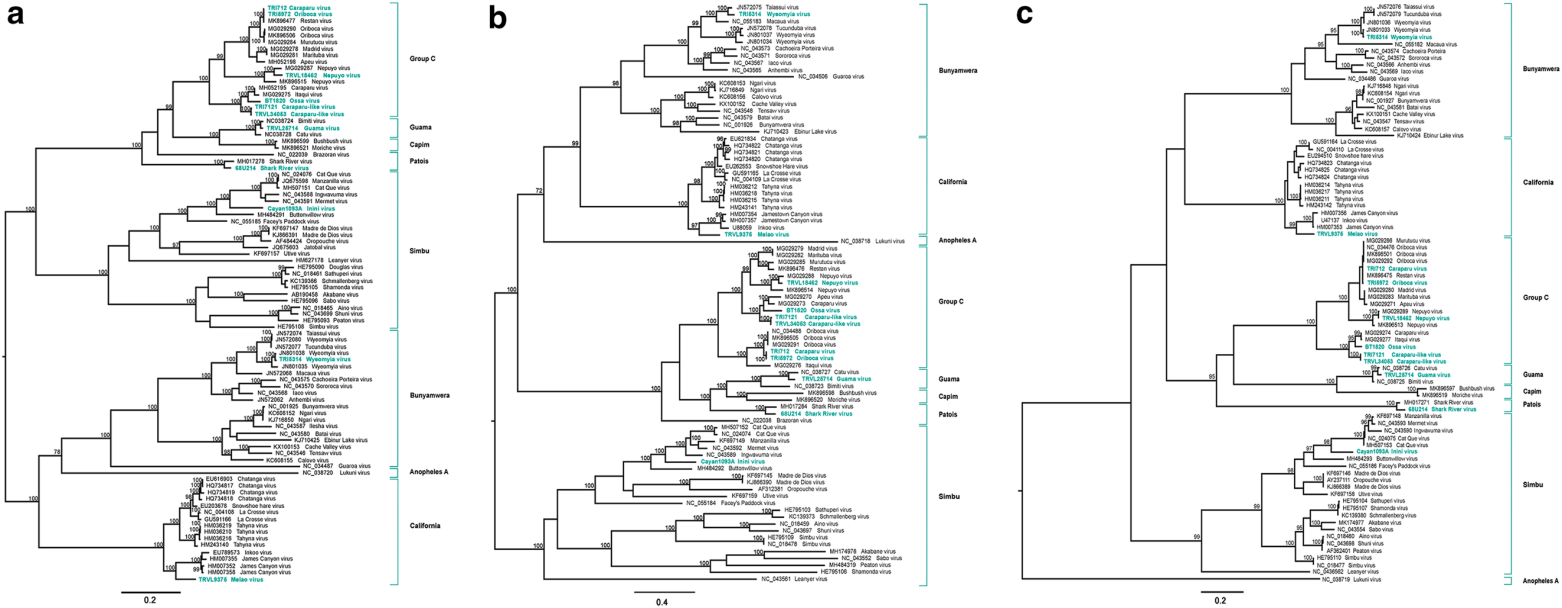
Phylogenetic analysis of the **a** L segment [polymerase], **b** M segment [membrane precursor polyprotein] and **c** S segment [nucleoprotein] of newly characterized *orthobunyavirus* strains and other representative members of the genus based on amino acid sequences deduced from the complete open reading frame sequences. Taxon labels include virus and strain or isolate designation and GenBank accession number. Analyses were carried out using maximum likelihood (ML) methods, yielding identical topologies, and the ML tree is presented. Statistical significance of the tree topology was evaluated by UFBoot resampling of the sequences 1000 times. UFBoot values greater than or equal to 95% and those associated with three apparent reassortant viruses are shown at relevant nodes. *Orthobunyavirus* sequences characterized in this study are indicated in teal

This study sought to increase the availability of complete genome sequences for an understudied genus of viruses, characterize the genetic diversity of viruses presently circulating in Trinidad, South and Central America, as well as confirm the serological identities of these isolates using sequence data. The 11 orthobunyaviruses characterized include representatives from six serogroups: two taxonomically unassigned viruses belonging to Group C, and nine viruses classified into Group C and five other serogroups (Table 1). The lengths of the predicted ORFs and proteins for all three genome segments of the sequenced strains were in close agreement with those previously reported for orthobunyaviruses (Online Resource 1) [26]. For the 11 orthobunyaviruses, classification based on amino acid phylogenies of all three genome segments was consistent with their historically assigned serogroup designations (Fig. 1). Among the represented serogroups, and for all three segments, the Group C virus sequences were most closely related to those of the Capim, Patois, and Guama serogroups, forming a distinct cluster of phylogenetic clades (UFBoot = 100% among all phylogenies in Fig. 1), while California, Simbu, and Bunyamwera serogroup sequences each formed separate and distinct clades.

Sequences for the Group C viruses Caraparu virus (CARV TRI712), Ossa virus (OSSAV BT1820), Oriboca (ORIV TRI5972), and Nepuyo virus (NEPV TRVL18462) consistently fell into the Group C clade along with the sequences of Caraparu-like virus (CARLV TRI7121 and TRVL34053), regardless of the segment analyzed. Among the Group C isolates from Trinidad, CARV TRI712 and ORIV TRI5972 consistently grouped together among all phylogenies (UFBoot = 100%), and CARLV strains TRI7121 and TRVL34053 similarly grouped together among all phylogenies (UFBoot = 100%). CARV TRI712, ORIV TRI5972, and CARLV TRI7121 viruses were all isolated from *Culex (Melanoconion) portesi* mosquitoes collected in Trinidad in 2007 and 2008. These data suggest the concurrent circulation of two distinct lineages of Group C viruses in Trinidad’s forests, both of which utilize the same vector *Culex (Melanoconion) portesi*. The amino acid identities of CARV TRI712 and ORIV TRI5972 across all three genome segments ranges from 99.5–99.7%, despite the two viruses being previously serologically identified to different species of the Group C complex (see Table 1). This sequence similarity confirms both viruses are the same species (i.e., likely ORIV) and further highlights the serological misclassification common among orthobunyavirus identification, and the importance of nucleotide sequencing in orthobunyavirus species identification.

Shark river virus (SRV 68U214), a member of the Patois serogroup, was most closely related and formed a distinct clade with SRV strain 64U80 that was isolated from Mexico in 1964 (Fig. 1). Wyeomyia virus (WYOV TR5314) fell in the Wyeomyia clade, while Inini virus (INIV Cayan1093A) fell into the Simbu clade in all three phylogenies. Guama virus (GMAV TRVL25714) consistently fell into the Guama clade in all three phylogenies. Melao virus (MELV TRVL9375) fell within the California clade as expected in all phylogenies.

Within the Group C serocomplex, Restan virus (RESV) strain TRVL 51144 (99.7–99.6% and 99.6–100%, respectively), which was isolated in 1963 in that same country (denoted as MK896477 & MK896475 in Fig. 1a and c) showed a strong phylogenetic relationship with high amino acid identity in their L & S segments with ORIV TRI5972 and CARV TRI712. However, comparisons of their M segments show that this RESV strain (denoted MK896476 Restan virus in Fig. 1b) exhibited only 66.3% and 66.1% amino acid identity with ORIV TRI5972 and CARV TRI712, respectively. Instead RESV showed greater phylogenetic relatedness (UFBoot = 100%) to Murutucu virus (MURV); another Group C virus isolated in 1955 from Brazil. Both RESV and MURV are serologically part of the Marituba species complex, within the Group C serocomplex (Table 1). This apparent repositioning of the RESV M segment sequence in the phylogeny, in contrast to the L and S segment phylogenies, provides evidence that this virus might be a natural reassortant. Similar reassortment events are known to occur naturally in orthobunyaviruses [10–15].

Additional patterns that could be attributed to reassortment were also observed in the Wyeomyia complex of viruses. The 1964 WYOV strain Darien from Brazil (JN801038 in Fig. 1a & JN103086 in Fig. 1c), and the 1955 WYOV TRVL8439 from Trinidad (JN108035 in Fig. 1a & JN 801033 in Fig. 1c) showed high similarity to the 2009 Trinidad strain WYOV TRI5314 in the L & S segments (UFBoot > 95). However, the M segment of WYOV TRI5314 was more closely associated with more recent Wyeomyia complex strains (UFBoot = 100%): Taiassui virus isolated in 1988, Brazil, and Macaua virus isolated in 1976, Brazil; for which the latter is thought to itself have undergone a reassortment [12]. As noted historically for the genera *Orthobunyavirus*, *Phlebovirus*, and *Hantavirus* (reviewed in [31–33]), the plausible reassortments described in this study involve the exchange of the M segment. This observation suggests a larger degree of compatibility factors such as packaging signals, RNA–RNA and/or RNA–protein interactions among the L and S segments’ sequences and gene products. The mechanisms underlying the permissiveness of this M segment exchange are still unknown, and future reverse genetics studies are needed to dissect the interactions that contribute to successful reassortment.

This study characterizes 11 orthobunyaviruses that have circulated in Trinidad and the wider South American region in the past five decades, addressing the paucity of available complete genome sequences for this increasingly important group of viruses. Our analyses also provide evidence for natural reassortment among strains circulating within Trinidad, via the M genome segment, which has been suggested to be the most likely reassortment event. The characterization of these arbovirus pathogens and other still partially characterized orthobunyaviruses is key in enabling the continued surveillance of strains that have the potential to become emergent pathogens in the future.

## Supplementary Information

Below is the link to the electronic supplementary material.

Supplementary file1 (DOCX 19 KB)

## Data Availability

All sequences derived in this study are available in GenBank. The sequence alignment data used in this study are available from the corresponding author upon request.
