# Supplementary material for: Phylogenetic characterization of Orthobunyaviruses isolated from Trinidad shows evidence of natural reassortment
Source: Virus Genes. 2023 Feb 10;59(3):473–8. doi: 10.1007/s11262-023-01973-5 (PMC10199832; doi:10.1007/s11262-023-01973-5)
Supplement: Supplementary file 1 — Supplementary file1 (DOCX 19 KB) [file 11262_2023_1973_MOESM1_ESM.docx]

**Title:** Phylogenetic characterization of Orthobunyaviruses isolated from Trinidad shows evidence of natural reassortment.

**Journal:** Virus Genes

**Author names:** Jerome E. Foster, Krisangel López, Gillian Eastwood, Hilda Guzman, Christine V. F. Carrington, Robert B. Tesh, Albert J. Auguste.

**Corresponding author address:** Department of Entomology, College of Agriculture and Life Sciences, Fralin Life Science Institute, Virginia Polytechnic Institute and State University, Blacksburg, VA, 24061; email: [jauguste@vt.edu](mailto:christine.carrington@sta.uwi.edu)

**Table S1. Lengths of open reading frames and predicted protein sequences for orthobunyaviruses sequenced in this study.**

| **Virus Species (Isolate/Strain)** | **Virus Genome Segment** | | | | | | | | |
| --- | --- | --- | --- | --- | --- | --- | --- | --- | --- |
|  | **L** | | | **M** | | | **S** | | |
|  | **Sequence Length (bp)** | **ORF length (position)** | **Protein length** | **Sequence Length (bp)** | **ORF length**  **(position)** | **Protein length** | **Sequence Length (bp)** | **ORF length**  **(position)** | **Protein length** |
| CARLV TRVL34053 | 6,888 | 6,747 (25-6771) | 2,248 aa | 4,557 | 4,305 (26-4330) | 1,434 aa | 1,089 | 318 (13-330)  708 (51-758) | 105 aa  236 aa |
| CARV  TRI712 | 6,747 | 6,747 (43-6789) | 2,248 aa | 4,557 | 4,305 (26-4330) | 1,434 aa | 1,083 | 318 (13-330)  708 (51-758) | 105 aa  236 aa |
| CARLV TRI7121 | 6,953 | 6,747 (51-6797) | 2,248 aa | 4,663 | 4,287 (66-4352) | 1,428 aa | 1,109 | 318 (37-354)  708 (75-782) | 105 aa  235 aa |
| GMAV TRVL25714 | 6,976 | 6,753 (56-6808) | 2,250 aa | 4,613 | 4,323 (55-4377) | 1,434 aa | 1,121 | 231 (468-698)  714 (58-771) | 76 aa  237 aa |
| INIV Cayan1093A | 6,854 | 6,786 (39-6824) | 2,261 aa | 4,437 | 4,287 (7-4293) | 1,428 aa | 964 | 309 (43-351)  702 (39-740) | 102 aa  233 aa |
| MELV TRVL9375 | 6,926 | 6,792 (51-6842) | 2,263 aa | 4,476 | 4,329 (52-4380) | 1,442 aa | 951 | 294 (85-378)  708 (66-773) | 97 aa  235 aa |
| NEPV TRVL18462 | 6,982 | 6,792 (5-6796) | 2,263 aa | 4,521 | 4,293 (35-4327) | 1,430 aa | 985 | 318 (32-349)  708 (70-777) | 105 aa  235 aa |
| ORIV  TRI5972 | 6,957 | 6,747 (43-6789) | 2,248 aa | 4,536 | 4,305 (19-4323) | 1,434 aa | 1,073 | 318 (6-323)  708 (44-751) | 105 aa  235 aa |
| OSSAV  BT1820 | 7,152 | 6,747 (67-6813) | 2,248 aa | 4,571 | 4,287 (26-4312) | 1,428 aa | 1,118 | 318 (25-342)  708 (63-770) | 105 aa  235 aa |
| SRV  68U214 | 6,945 | 6,744 (37-6780) | 2,247 aa | 4,575 | 4,293 (13-4305) | 1,430 aa | 1,014 | 390 (26-415)  711 (61-771) | 129 aa  236 aa |
| WYOV  TRI5314 | 6,798 | 6,711 (33-6743) | 2,236 aa | 4,509 | 4,257 (20-4276) | 1,418 aa | 1,136 | 390 (626-237)  702 (78-779) | 130 aa  233 aa |
|  |  |  |  |  |  |  |  |  |  |
| Bunyamwera virus* | 6,875 | 6,717 (51-6767) | 2,238 aa | 4,458 | 4,302 (57-4358) | 1,486 aa | 961 | 306 (105-410)  702 (86-787) | 101 aa  234aa |
| Cache Valley virus* | 6,871 | 6,717 (42-6758) | 2,239 aa | 4,460 | 4,305 (45-4349) | 1,435 aa | 905 | 306 (789-384)  702 (60-761) | 102 aa  234 aa |
| La Crosse virus* | 6,980 | 6,792 (62-6853) | 2,264 aa | 4,527 | 4,326 (62-4837) | 1,442 aa |  | 279 (101-379)  708 (72-789) | 93 aa  236 aa |
| Oropouche virus* | 6,852 | 6,759 (44-6802) | 2,253 aa | 4,385 | 4,263(32-4294) | 1,421 aa | 958 | 276 (67-342)  696 (45-740) | 92 aa  232 aa |

*Indicates prototype *orthobunyavirus* species. Bunyamwera virus sequences for L, M, & S obtained from GenBank accession numbers NC_001925.1, NC_001926.1 & NC_001927 respectively (Lees et al, 1986); Cache Valley virus sequences for L, M, & S obtained from GenBank accession numbers KC436106.1, KC436107.1 & KC436108.1 respectively (Nyugen et al, 2013); La Crosse virus sequences for L, M & S obtained from GenBank accession numbers NC_004108.1, NC_004109.1 & NC_004110.1 respectively; Oropouche virus sequences for L, M & S obtained from GenBank accession numbers KP052850.1, KP052851.1 & KP052852.1 respectively.
